# Gene therapy: an emerging therapy for hair cells regeneration in the cochlea

**DOI:** 10.3389/fnins.2023.1177791

**Published:** 2023-05-03

**Authors:** Jipeng Wang, Jianwei Zheng, Haiyan Wang, Haoying He, Shuang Li, Ya Zhang, You Wang, Xiaoxiang Xu, Shuyi Wang

**Affiliations:** ^1^Department of Gastrointestinal Surgery, Zhongnan Hospital of Wuhan University, Wuhan, China; ^2^Department of Biliary-Pancreatic Surgery, Tongji Hospital, Tongji Medical College, Huazhong University of Science and Technology, Wuhan, China; ^3^Department of Otolaryngology-Head and Neck Surgery, Zhongnan Hospital of Wuhan University, Wuhan, Hubei, China; ^4^Department of Neurology, Zhongnan Hospital of Wuhan University, Wuhan, Hubei, China; ^5^Department of Stomatology, Zhongnan Hospital of Wuhan University, Wuhan, Hubei, China

**Keywords:** hair cell regeneration, gene therapy, *Atoh1*, inner ear, sensorineural hearing loss

## Abstract

Sensorineural hearing loss is typically caused by damage to the cochlear hair cells (HCs) due to external stimuli or because of one’s genetic factors and the inability to convert sound mechanical energy into nerve impulses. Adult mammalian cochlear HCs cannot regenerate spontaneously; therefore, this type of deafness is usually considered irreversible. Studies on the developmental mechanisms of HC differentiation have revealed that nonsensory cells in the cochlea acquire the ability to differentiate into HCs after the overexpression of specific genes, such as *Atoh1*, which makes HC regeneration possible. Gene therapy, through *in vitro* selection and editing of target genes, transforms exogenous gene fragments into target cells and alters the expression of genes in target cells to activate the corresponding differentiation developmental program in target cells. This review summarizes the genes that have been associated with the growth and development of cochlear HCs in recent years and provides an overview of gene therapy approaches in the field of HC regeneration. It concludes with a discussion of the limitations of the current therapeutic approaches to facilitate the early implementation of this therapy in a clinical setting.

## 1. Introduction

Deafness is the most common neurological disorder in humans, which has seriously affected the normal life of human beings. According to the World Hearing Report published by the World Health Organization, almost 1.5 billion people worldwide have varying degrees of hearing loss, and 430 million people are at a level of severe hearing loss that requires rehabilitation (Chadha et al., 2021). Deafness can be categorized as conductive, sensorineural, and mixed deafness (Cunningham and Tucci, 2017). The more common type of deafness is sensorineural deafness caused by death or functional loss of cochlear hair cells (HCs). HCs are the most critical cells for sound perception and transmission in the inner ear sensory cells, and their function is to convert the mechanical signals of sound coming in from the environment into electrical signals that the nervous system can perceive (Deans, 2021). HCs are the most critical cells in the mammalian inner ear sensory epithelium. Studies have shown that (Fujioka et al., 2015) compared with nonmammals (birds and reptiles), HCs cannot regenerate spontaneously in mammals; thus, HC damage often results in permanent hearing loss.

Gene therapy involves transferring an external normal or therapeutic gene, via a vector, to a target cell in the body, causing the target cell to express the relevant gene or to modify the pertinent gene as a therapeutic approach. It has now become a potential treatment for genetic deafness. In several animal models, gene therapy has been used to transfer several genes such as *Syne4* (Taiber et al., 2021), Tmc1 (Marcovich et al., 2022), and *Clarin-1* (Dulon et al., 2018) moved into the cochlea and has significantly improved the degree of hearing impairment in the study animals. During the developmental differentiation and maturation of inner ear HCs, there is also the regulation of multiple genes (Bermingham et al., 1999; Hertzano et al., 2004; Ikeda et al., 2015; Hou et al., 2019; Ding et al., 2020; Jen et al., 2022) and signaling pathways (Benito-Gonzalez and Doetzlhofer, 2014; Waqas et al., 2016; Ebeid and Huh, 2017; Bai et al., 2021). By interfering with these, the normal differentiation of HCs can be restored, and support cells (SCs) can be stimulated to re-differentiate and produce HCs (Menendez et al., 2020). The aim is to treat hearing loss associated with HC damage. In this review, we highlight how gene therapy can promote hair cell regeneration as a way to alleviate the hearing loss in patients and provide an outlook for future research in this area.

## 2. HC development–related transcription factors

**Figure 1:**
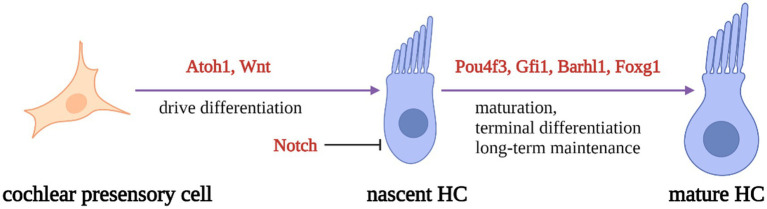
Schematic diagram of HC development process. Regulatory factors Atoh1 and Wnt signaling pathways are necessary for presensory cells to differentiate into initial HCs. Atoh1 downstream targeting factors (Pouf4, Gfi1, Barhl3) and Foxg1 play essential roles in nascent HCs maturation and long-term maintenance. At the same time, Notch signal pathway can inhibit the expression of Atoh1 in presensory cells and regulate the differentiation of HCs.

**Figure 2:**
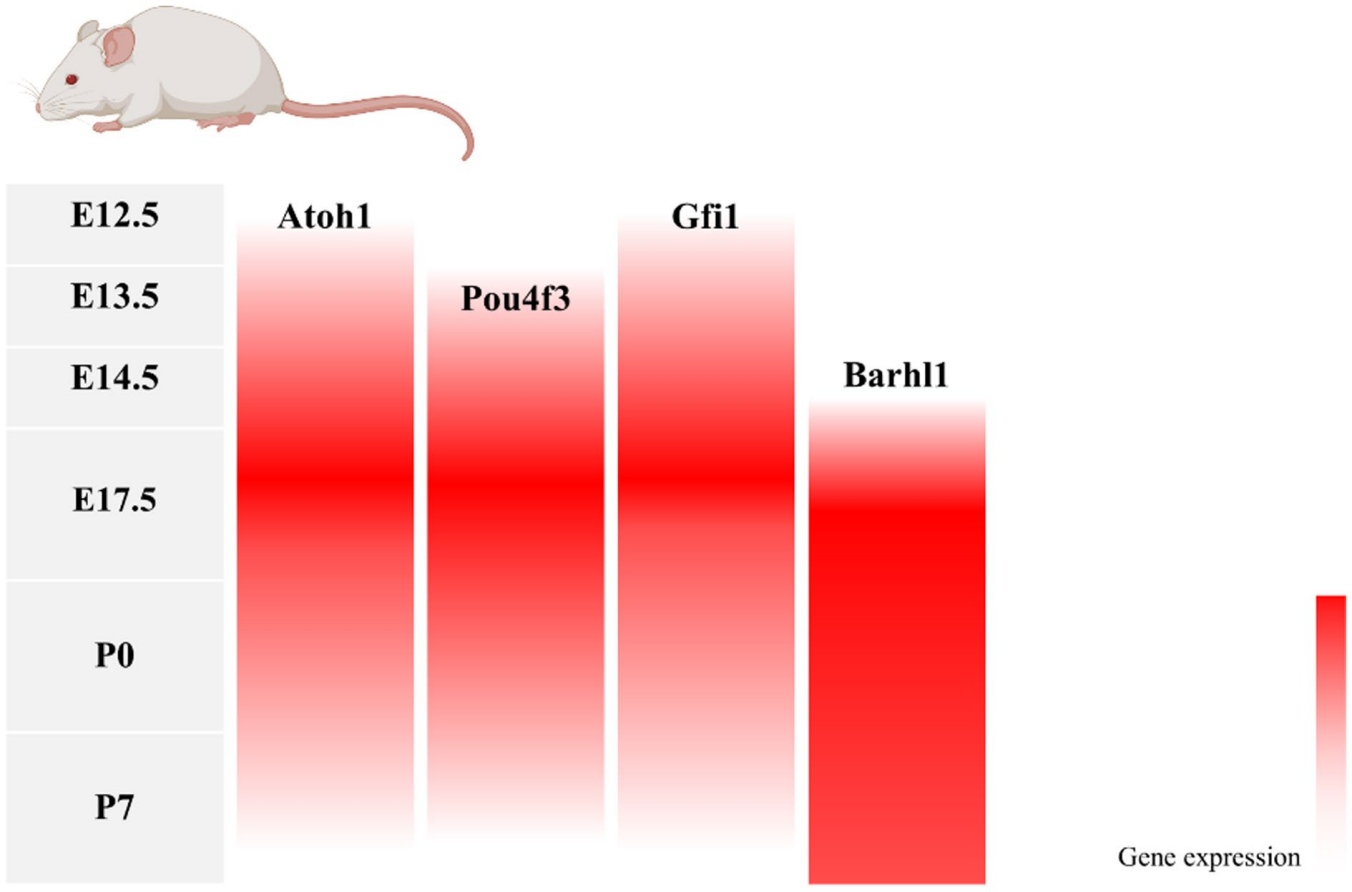
Changes in gene expression with age in a mouse model of inner ear development. During mouse embryonic cochlea development, *Atoh1* and its downstream target transcription factors *Pou4f3*, *Gfi1*, and *Barhl1* were successively expressed, with *Atoh1*, *Pou4f3*, and *Gfi1* decreasing in expression after birth as the cochlea matured, and *Barhl1* continuing to be expressed.

During inner ear development, many transcription factors, including *Atoh1*, are involved in the proliferation and differentiation of HCs (Figure 1). In a mouse model of inner ear development, *Atoh1* was first expressed in the basal progenitor HCs at embryonic stage (E) 13.5 d, and gradually increased until the cochlear spiral matured at E17.5, and gradually decreased after postnatal (P) 0 d. After P7, *Atoh1* expression could not be measured in the spiral (Lumpkin et al., 2003; Cotanche and Kaiser, 2010; Cai et al., 2013). In contrast, the change of *Atoh1*-related downstream targeting factor *Gfi1* was consistent with the change of *Atoh1*, which started to be expressed at E12.5 and also gradually decreased in expression with the end of embryonic stage (Wallis et al., 2003). Conversely, *Pou4f3* and *Barhl1* were detected in cochlear basal HCs only at E13.5 and E14.5, respectively, and continued to be expressed after birth (Xiang et al., 1997; Hou et al., 2019; Figure 2).

### 2.1. Atoh1

*Atoh1*, also known as *Math1*, is a helix–loop–helix (bHLH) family transcription factor with a coding sequence of 1.053 kb, encoding a protein of size 17.9 kDa. *Atoh1* was the first transcription factor identified in differentiated HC progenitors and is essential for HCs growth and differentiation (Bermingham et al., 1999). In *Atoh1* mutant mice, all inner ear sensory regions do not differentiate to produce HCs (Pan et al., 2011). Further studies revealed that the dependence of HCs on *Atoh1* diminishes as sensory cells in the cochlea develop and mature (Chonko et al., 2013). However, it is not the case that the HCs are unaffected by *Atoh1* after cochlear growth, as *Atoh1* deficiency also disrupts the standard hair bundle structure of the auditory system and eventually leads to the delayed death of HCs (Cai et al., 2013; Cheng et al., 2016). In contrast, the enhanced expression of *Atoh1* promotes the normal development of HCs and improves hearing (Izumikawa et al., 2005; Luo et al., 2022). Thus, the entire auditory system, from the developmental to mature stages, is inseparable from the regulation of *Atoh1*.

### 2.2. *Atoh1* downstream targeting factors *Pou4f3*, *Gfi1*, and *Barhl1*

Due to the importance of *Atoh1* in HCs, identifying the downstream targeting factors of *Atoch1* is crucial to investigate developmental mechanisms. *Atoh1* target groups were identified in mouse cerebellum and cochlea development was studied using genome-wide *Atoh1* sequencing methods (Klisch et al., 2011; Cai et al., 2015). The direct *Atoh1* target genes *Pou4f3*, *Gfi1*, and *Barhl1* are associated with the normal differentiation and regeneration of HCs (Wallis et al., 2003; Zhong et al., 2018; Chen et al., 2021). The *Atoh1* target group has been identified in the cochlea.

*Pou4f3*, a Pou family transcription factor, is the dominant nonsyndromic deafness 15 (DFNA15) deafness-causative gene (Vahava et al., 1998) and a downstream target of *Atoh1* activation (Ikeda et al., 2015). During HC differentiation, there is a feed-forward synergy between *Atoh1* and *Pou4f3*, with *Atoh1* first stimulating *Pou4f3* expression, which releases *Atoh1*-related elements in a closed state to activate a series of HC-specific enhancers (Yu et al., 2021). *Gfi1* is a zinc-finger transcription factor. Studies have shown that *Gfi1* expression is regulated by *Pou4f3* (Hertzano et al., 2004). *Gfi1* represses neuronal gene expression early in the development of HCs, and in the absence of *Gfi1*, cochlear maturation is stalled (Matern et al., 2020). *Barhl1* is a BarH-like homologous domain transcription factor explicitly expressed in all HCs in the cochlear (Bulfone et al., 2000). Mice lacking *Barhl1* developed severe age-related hearing loss. Further studies have found that HC death in *Barhl1*-null mice begins after 6 days of life and progresses slowly over several months (Li et al., 2002), suggesting that *Barhl1* may be involved in the terminal differentiation and long-term maintenance of HCs.

In conclusion, *Atoh1* is a crucial transcription factor in the formation of HCs, and *Atoh1* mutants lose the ability to generate HC progenitors; *Pou4f3* and *Gfi1*, the genes downstream of *Atoh1*, are required for the late developmental maturation of progenitors into HCs, and delayed degeneration of HCs occurs in *Pou4f3* and *Gfi1* mutants; *Barhl1* is associated with the long-term maintenance of HCs. In *Barhl1* mutants, HCs mature but eventually die within a certain period.

### 2.3. Foxg1

*Foxg1*, a member of the FOX family, is known to regulate ATP synthesis and metabolism in mitochondria (Pancrazi et al., 2015). *Foxg1* is essential for proper development and formation of the inner ear. In *Foxg1*-null mice, severe inner ear malformations, including shortened cochleae with multiple rows of HCs and supporting cells and reduced or even absent cristae have been reported (Pauley et al., 2006; Hwang et al., 2009). Mechanistically, deletion of *Foxg1* causes inhibition of Notch, Wnt, IGF, and EGF signaling pathways, production of HCs, and induction of their subsequent apoptosis (He et al., 2019). In addition, *Foxg1* regulates auditory degeneration through the regulation of autophagy. In the *Foxg1* downregulated group, the autophagic pathway was significantly inhibited, and reactive oxygen species levels were significantly increased, ultimately leading to the apoptosis of HCs (He et al., 2021). Similarly, *Foxg1* downregulation also considerably increased the sensitivity of HCs to lipopolysaccharide-induced inflammation and accelerated the apoptosis of HCs under inflammatory conditions (He et al., 2020).

## 3. Gene therapy promotes the regeneration of HCs

### 3.1. Gene therapy targets for the regeneration of HCs

**Figure 3:**
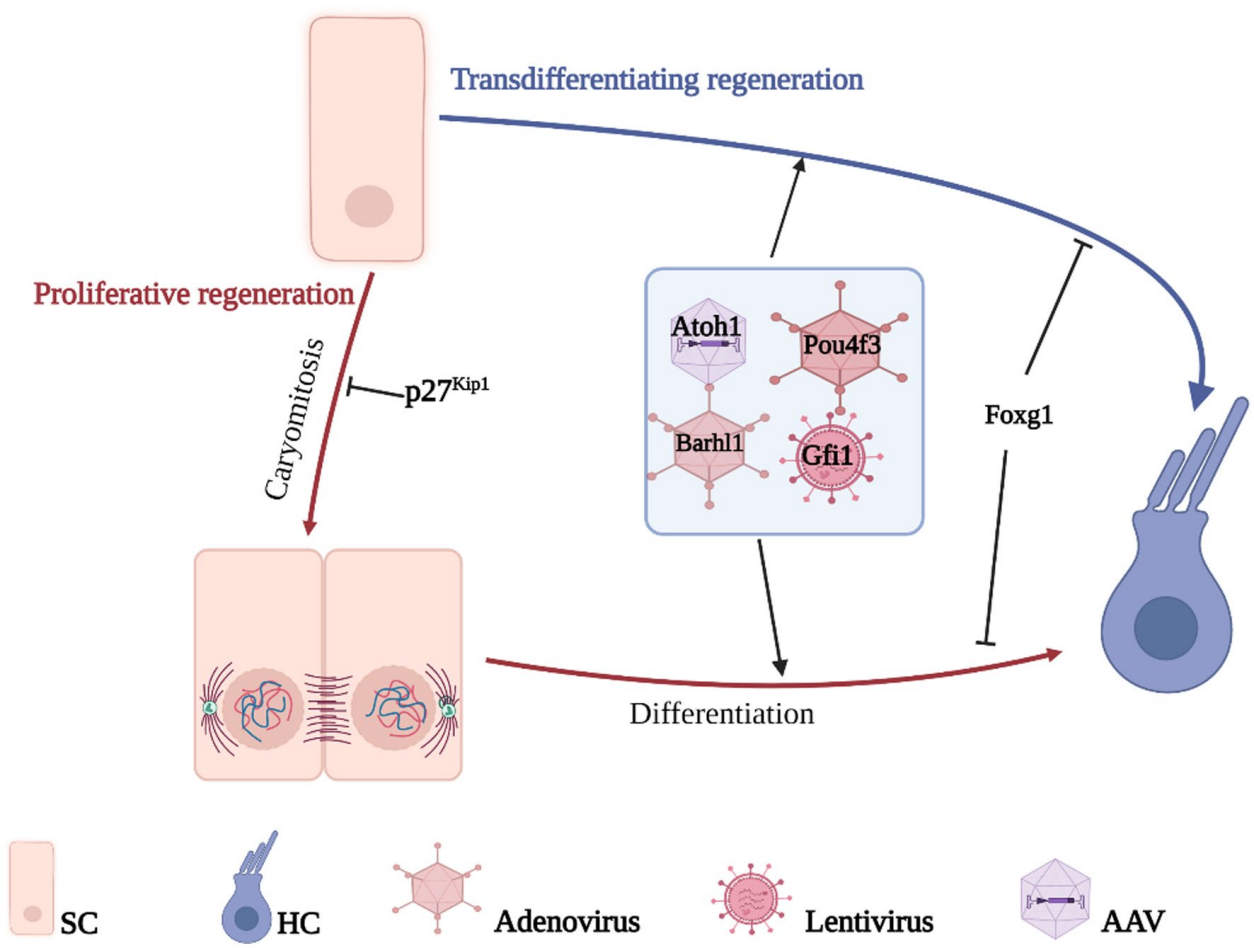
Gene therapy promotes HCs regeneration. HCs regenerate mainly through two pathways, direct transdifferentiation of SCs or proliferative differentiation of SCs, the difference between the two is whether mitosis is performed or not. The genes related to the growth, development, and maturation of HCs are transferred to the target cells by gene vector so that the non-sensory cells in the cochlea can differentiate into HCs and realize the regeneration of HCs.

Due to the critical role that individual genes play in the differentiation and development of HCs, developmental failure during HC differentiation occurs after the deletion of relevant genes. Therefore, inducing re-differentiation to generate new HCs by reprogramming HC-related genes in the inner ear SCs is a potential way to improve HC-related hearing impairment (Costa et al., 2015; Ni et al., 2016; Shibata et al., 2020). HCs regenerate mainly through two pathways: one activates non-sensory cell activation to re-enter the cell cycle and further divide and differentiate into HCs; the other directly induces non-sensory cells to transdifferentiate into HCs without mitosis (Figure 3).

#### 3.1.1. HCs proliferative regeneration

Cell cycle inhibitors are critical for maintaining cells in a quiescent state after mitosis, and therefore activation of non-sensory cells to re-enter the cell cycle requires regulation of the corresponding inhibitors. *P27^Kip1^ (p27)*, a member of the Cip/Kip family of cell cyclin-dependent protein kinase inhibitors, is significantly upregulated in dormant cells (Bencivenga et al., 2021) and has been shown to be a common cell cycle inhibitor for sensory and non-sensory cells in the inner ear (Chen and Segil, 1999; Löwenheim et al., 1999). Knocking down *p27* in isolated mouse cochlear cells can effectively activate the proliferation of SCs in cochlea to re-enter the cell cycle, and mitotically generated SCs retain the ability to redifferentiate into HCs (Löwenheim et al., 1999; White et al., 2006; Ono et al., 2009; Maass et al., 2013). Further studies revealed that in *p27* knockout mice, more than just SCs broke out of cell cycle quiescence, HCs also gained some proliferative capacity (Walters et al., 2014), and similar effects were achieved using Retinoic acid to inhibit *p27* (Rubbini et al., 2015). Combined with p27 knockdown, the transdifferentiation of *Atoh1* to produce HCs is not limited to the embryonic period and enables the regeneration of HCs in the mature mouse cochlea (Walters et al., 2017). Unfortunately, the production of HCs by mitotic re-differentiation of SCs does not function properly in mammals, but *p27* remains a potential target for the regeneration of cochlear HCs.

#### 3.1.2. HCs transdifferentiating regeneration

*Atoh1*, the first HC development–related transcription factor to be identified, plays an irreplaceable role in HC regeneration. In *ex vivo* experiments in normal rats and guinea pigs, *Atoh1* overexpression enabled nonsensory cells of the cochlea to acquire the ability to produce new HCs (Kawamoto et al., 2003; Shou et al., 2003). In a guinea pig model of deafness generated by ototoxic drug-induced HC death, *Atoh1* was injected into the cochlea of deaf animals via an adenoviral vector to increase its expression in nonsensory cells, showing that new HCs were produced at the original site of cochlear trauma. Hearing was restored to some extent in deaf animals as measured based on the auditory brainstem response (ABR) thresholds (Izumikawa et al., 2005). The results showed that new HCs were produced at the original site of trauma in the cochlea and that deaf animals had some hearing recovery as measured using the ABR thresholds.

In contrast, in a model of aminoglycoside-induced profound deafness, although *Atoh1* gene therapy induced the conversion of nonsensory cells in the cochlea into HCs, the resulting HCs failed to mature fully and did not improve hearing in the treated animals (Atkinson et al., 2014). This finding suggests that a combination of gene therapy modalities is required to maximize hearing function in patients. In the Mouse embryonic stem cells cultured *in vitro*, various transcription factors (*Six1*, *Atoh1*, *Pou4f3*, and *Gfi1*) reprogrammed mouse embryonic fibroblasts and expressed the corresponding HC markers. The resulting HCs that were induced were morphologically and physiologically similar to and susceptible to ototoxic drugs as in the case of primary HCs (Costa et al., 2015; Menendez et al., 2020). Similarly, the overexpression of *Gfi1*, *Pou4f3*, and *Atoh1* in human fibroblasts resulted in cells expressing some markers of HCs (Duran Alonso et al., 2018). In drug-treated mouse cochlear sensory epithelial cells, the damage caused by HC death can be reversed by the cotransfection of *Pax2* and *Atoh1*, with *Pax2* promoting the proliferation of SCs and *Atoh1* promoting the regeneration of HCs (Chen et al., 2013). In addition, HC-like cells were generated 4.1-fold more efficiently after cotransfection with *Atoh1* and *Gfi1* than with *Atoh1* alone (Lee et al., 2020); *Atoh1* and *Ikzf2* overexpression induced the transformation of SCs into cochlear outer HCs in the adult mouse cochlea (Sun et al., 2021). The expression of *Atoh1*, *Gfi1*, and *Pou4f3* increased the potency of HC transformation in aged animals (Iyer et al., 2022).

Wnt and Notch pathways play an essential role in cell proliferation and differentiation, including regulating HC differentiation in the cochlea (Ni et al., 2016; Waqas et al., 2016; Wu et al., 2016; Samarajeewa et al., 2019). Disruption of the *Rbpsuh* gene in neonatal mice or treatment of mouse inner ear cells with γ-secretase inhibitor resulted in inhibition of Notch/RBP-J pathway signaling, which in turn led to downregulation of *Hes5* expression and upregulation of *Atoh1* expression, ultimately producing ectopic HCs (Yamamoto et al., 2006; Mizutari et al., 2013; Ren et al., 2016; Luo et al., 2017). Using siRNA to downregulate *Hes1/Hes5* can also achieve *Atoh1* upregulation and increase the efficiency of conversion of HCs by SCs (Du et al., 2013; Jung et al., 2013). Adenovirus carrying human *Myc* and *Cre* recombinase genes was injected into the cochlea of adult mice, and an increase in HC numbers was observed along with the inhibition of the Notch pathway (Shu et al., 2019). The expression patterns of the hypermethylated 1 (*HIC1*) transcriptional repressor and *Prox1* genes do not overlap with *Atoh1* and related downstream genes, and studies confirm that both have a repressive effect on *Atoh1* and are responsible for the decrease in *Atoh1* expression in postnatal mice, whereas knockdown of *HIC1* or *Prox1* reverses the repression of *Atoh1* expression and ultimately promotes the differentiation of HCs (Kirjavainen et al., 2008; Abdul-Aziz et al., 2021). Meanwhile, an increase in *Atoch1* expression was induced by the *Atoch1* enhancer or small activating RNA to regulate HC regeneration (Luo et al., 2022; Zhang et al., 2022).

Moreover, recent studies on *Foxg1* have demonstrated its potential as a new target for the regeneration of HCs using gene therapy. In mice with *Foxg1* was knocked out in the inner ear SCs, HC numbers were significantly increased compared to those in normal mice, and the survival time was greatly increased (Zhang Y. et al., 2020; Zhang S. et al., 2020).

In conclusion, with a clear understanding of the mechanism of developmental differentiation of HCs, the regeneration of HCs can be achieved by interference with the relevant genes and pathways, thus reversing hearing loss caused by HC damage.

### 3.2. CRISPR/Cas9 gene editing system

**Figure 4:**
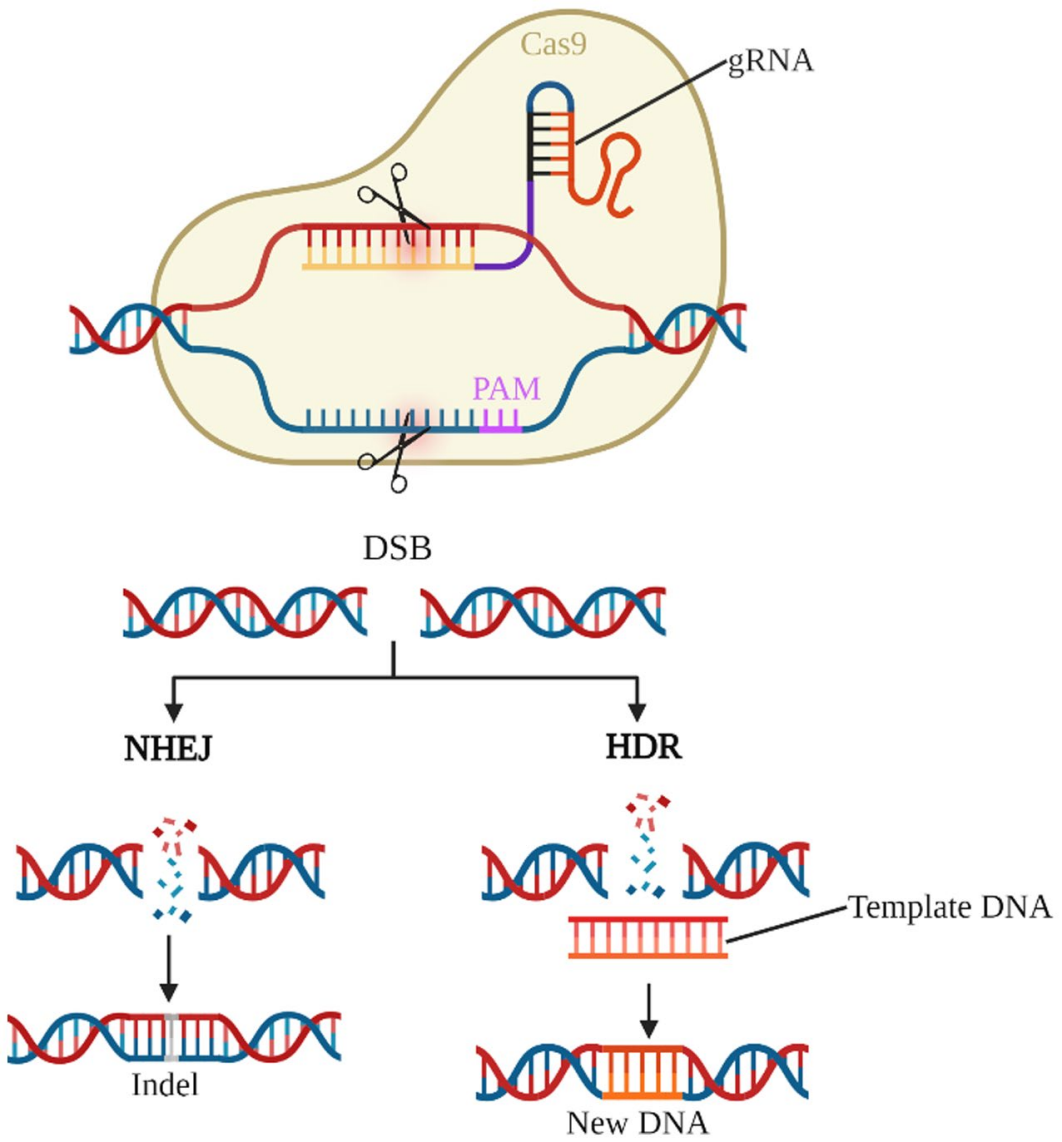
CRISPR/Cas9 gene editing system. The gRNA-Cas9 complex enters the cell and identifies the target gene corresponding to the PAM and shears it into a DSB. NHEJ causes the target gene shift mutation to achieve gene knock-out. HDR, on the other hand, repairs the target gene according to the exogenous template DNA and completes the gene knock-in.

As the third generation of gene editing technology after zinc-finger nucleases (ZFNs) and transcription activator-like effector nucleases (TALENs), the CRISPR/Cas system has the advantages of clear targeting, short RNA sequences, and simultaneous operation of multiple genetic loci, of which the type II CRISPR/Cas9 system is the most widely applied (Bhatia et al., 2023; van der Oost and Patinios, 2023; Wang and Doudna, 2023). Cas9 protein with nucleic acid endonuclease function and single guide RNA (sgRNA) shear the target genome to generate double-strand breaks (DSB), which in turn enables knockdown or knock-in of the target gene by Homology-directed repair (HDR) or Non-homologous end joining (NHEJ) to achieve knockdown or knock-in of target genes (Figure 4). Previous studies have used the CRISPR/Cas9 system to establish transgenic mouse models of deafness to investigate the importance of target genes for the development and maintenance of normal hearing in the inner ear HCs (Li et al., 2018, 2019; Zhu et al., 2018; Cui et al., 2020; Zhang L. et al., 2020; Tu et al., 2021; Xue et al., 2022). CRISPR-Cas9 technology is now also showing great potential in clinically blocking dominant and recessive mutations in deafness and improving hearing impairment (György et al., 2019; Farooq et al., 2020; Ding et al., 2021).

Beethoven deaf mice are deafened by a point mutation (T into A) in the *Tmc1* gene at locus 1,235, causing hearing impairment associated with reduced HCs in the inner ear and successfully targeting the *Tmc1* gene by the lipid-mediated entry of the Cas9-gRNA complex into the mouse cochlea, resulting in a shift mutation and eventual loss of function due to a random insertion–deletion, which improves the survival of HCs while improved the hearing of mice (Gao et al., 2018). Efficient knockdown of the *Htra2* gene associated with apoptosis by transfection of three gRNAs into *in vitro* cochlear explants and *in vivo* scala medium *via* the CRISPR/Cas9 system improved hearing loss caused by neomycin-induced hair cell apoptosis (Gu et al., 2021). In addition, CRISPR-Cas9 knockdown of *Kcnq4* and myosin VI (*MYO6*) mutant genes have been shown to rescue inherited hearing impairment (Noh et al., 2022; Xue et al., 2022).

Although the CRISPR/Cas9 system can accurately and efficiently edit target genes, it also has limitations in hearing damage rescue studies. The presence of a short DNA sequence called the pro-spacer adjacent motif (PAM) near the complementary region of the gRNA and the target gene. The PAM sequences are mainly used to identify targets, and the presence or absence of PAM sequences in target nucleotides is a critical factor in the precise targeting of the CRISPR/Cas9 system (Manghwar et al., 2019).

### 3.3. Gene delivery vectors

The two main types of vectors for gene therapy are viral vectors and non-viral vectors, namely, viral vectors including adenovirus (AdV), adeno-associated virus (AAV), lentivirus, and retroviruses; and nonviral vectors including electroporation, liposomes, nanoparticles, and exosomes (Cring and Sheffield, 2022). The choice of gene therapy vector is significant, as it needs to deliver the exogenous gene safely and effectively to the cells of the inner ear without causing a robust immune response and to sustain its action.

AdVs were the first gene delivery vectors to be used; they are now used in various fields for HC regeneration (Syyam et al., 2022). *In vivo* or *in vitro* experiments involving the injection of AdVs carrying different target genes into target cell tissues can effectively transduce nonsensory cells into HCs, with SCs being the main ones transduced (Kawamoto et al., 2003; Shou et al., 2003; Izumikawa et al., 2005; Yamamoto et al., 2006; Chen et al., 2013; Atkinson et al., 2014; Shu et al., 2019; Lee et al., 2020). However, AdVs have a significant immunogenic effect, and their role in gene therapy is somewhat limited. In contrast, AAVs have a much lower immunoreactivity and have gradually become the vehicle of choice for gene therapy in different fields. AAVs have demonstrated their safety and efficacy in gene therapy for the regeneration of HCs. Injecting AAV8 in normal neonatal and adult mice did not cause damage to HCs in the inner ear or hearing loss (Kang et al., 2020). In addition, AAV-mediated gene delivery effectively ameliorates apoptosis and hearing loss of HCs in a drug-induced mouse model of deafness in the long term (Brigande et al., 2009; He et al., 2020; Gu et al., 2021; Xu et al., 2021; Xue et al., 2022). The results of this study are summarized below. Recent studies have shown that AAV-inner ear, a variant of AAV, can more safely and efficiently transduce Atoh1 into SCs and may be the best vehicle for future gene therapy to combat hearing loss (Tan et al., 2019; Tao et al., 2022). In addition, lentiviruses and retroviruses can also be used to deliver HC regeneration–related genes; however, their safety profile needs to be improved (Costa et al., 2015; Menendez et al., 2020).

Other nonviral gene delivery methods have also been used to regenerate HCs. *Hes1* siRNA delivered by propylene-co-glycolate nanoparticles can reduce cochlear *Hes1* mRNA while upregulating *Atoh1* mRNA expression and, in doing so, promote the ability of SCs to acquire redifferentiated HCs (du et al., 2013). In addition, electroporation was influential in transducing plasmids encoding target genes such as *Tub* and *Znf532* into the epithelial progenitor cells of the ear, activating the regeneration of HCs mediated by genes such as *Atoh1* (Brigande et al., 2009; Xu et al., 2021).

## 4. Summary and perspectives

HCs in the cochlea, as key members of the auditory conduction system, transform incoming mechanical signals into electrical signals for the body to perceive. They do not regenerate spontaneously in mammals, resulting in the associated hearing impairment being poorly treated. An exploration of the developmental maturation mechanisms of HCs reveals that the HC regulatory gene *Atoh1* and its downstream targeting factors activate the ability of nonsensory SCs to differentiate into HCs. Emerging gene therapies can deliver external DNA or RNA into target cells via vectors to alter the gene expression of target cells and improve relevant functions. After addressing congenital hearing impairment, gene-based therapies can be used to treat other types of hearing impairment with the help of HC regeneration mechanisms (Table 1).

**Table 1:** Summary of studies on regenerative gene therapy for HCs.

Experimental subjects	Mode of gene transfer	Contents	Results	References
A model of ototoxic drug-induced deafness in guinea pigs	Adenovirus	*Atoh1*	HCs regenerate, and hearing improves.	Kawamoto et al. (2003), Shou et al. (2003), Izumikawa et al. (2005), and Atkinson et al. (2014)
Mouse embryonic stem cells	Retrovirus/Lentivirus	*Six1 Atoh1 Pou4f3 Gfi1*	Produces induced HCs similar to HCs	Costa et al. (2015) and Menendez et al. (2020)
Human fibroblasts	Lentivirus	*Atoh1 Pou4f3 Gfi1*	Acquisition of cells expressing markers of HCs	Duran Alonso et al. (2018)
Cochlear sensory epithelium in mice after neomycin injury	Adenovirus	*Pax2 Atoh1*	Inducing the production of new HCs with some functional activity	Chen et al. (2013)
Pou4F3^DTR^ mice after diphtheria toxin injury	Adenovirus	*Atoh1 Gfi1*	Induced production of HC-like cells is more efficient than *Atoh1* alone	Lee et al. (2020)
Exosomes of mouse Corti organs carrying the Rbpsuh allele	Adenovirus	Cre recombinase gene	*Rbpsuh* target genes were deleted to obtain cells expressing HC markers	Yamamoto et al. (2006)
A model of ototoxic drug-induced deafness in mice	Physiological saline/propylene-co-glycolic acid nanoparticles	*Hes1/Hes5* siRNA	Upregulation of *Atoh1* expression and increase in the number of HCs	du et al. (2013) and Jung et al. (2013)
Rosa-NICD transgenic mice	Adenovirus	*Myc* and *Cre* recombinase genes	Notch pathway inhibition, HC numbers rise	Shu et al. (2019)
C57BL / 6 mice	AAV-ie	*Atoh1*	A large number of infantile HCs appeared compared with the control group	Tan et al. (2019) and Tao et al. (2022)
FVB mice	Electroporation	*Tub Znf532*	Promotes *Atoh1*-mediated regeneration of HCs while ensuring minimal damage to endogenous HCs	Xu et al. (2021)

Many challenges remain in inducing regeneration of HCs in clinical settings based on gene therapy. First, the growth and development of HCs are regulated by multiple genes and pathways, and a single gene alone cannot bring about the differentiation of SCs into fully functional mature HCs. Second, the choice of vectors for gene delivery is also essential, as it is necessary to deliver the gene to the target cells efficiently and accurately without inducing a robust immune response in the body. Finally, enhancing the efficiency of HCs regeneration while ensuring high targeting requires innovation in multiple steps of gene therapy. Recent studies using the CRISPR/Cas9 system in combination with AAV vectors have shown great advantages (Kang et al., 2020; Zhao et al., 2020).

In conclusion, HC regeneration–based gene therapy shows immense potential in treating sensorineural hearing impairment. It is expected to be used in a clinical setting after further research on the mechanism of HC regeneration and optimizing targeted gene delivery methods.

## Author contributions

All authors listed have made a substantial, direct, and intellectual contribution to the work and approved it for publication.

## Funding

This work was supported in part by grants from the National Natural Science Foundation of China (Grant No. 82271181) and Zhongnan Hospital of Wuhan University Science, Technology and Innovation Seed Fund, Project CXPY2022092.

## Conflict of interest

The authors declare that the research was conducted in the absence of any commercial or financial relationships that could be construed as a potential conflict of interest.

The reviewer YZ declared a shared parent affiliation with the authors JW, HW, HH, SL, YZ, YW, XX, and SW at the time of review.

## Publisher’s note

All claims expressed in this article are solely those of the authors and do not necessarily represent those of their affiliated organizations, or those of the publisher, the editors and the reviewers. Any product that may be evaluated in this article, or claim that may be made by its manufacturer, is not guaranteed or endorsed by the publisher.
